# Single-Cell Technologies to Decipher the Immune Microenvironment in Myeloid Neoplasms: Perspectives and Opportunities

**DOI:** 10.3389/fonc.2021.796477

**Published:** 2022-02-02

**Authors:** Chiara Caprioli, Iman Nazari, Sara Milovanovic, Pier Giuseppe Pelicci

**Affiliations:** ^1^ Department of Experimental Oncology, IRCCS Istituto Europeo di Oncologia, Milan, Italy; ^2^ Scuola Europea di Medicina Molecolare (SEMM) European School of Molecular Medicine, Milan, Italy; ^3^ Hematology and Bone Marrow Transplant Unit, Papa Giovanni XXIII Hospital, Bergamo, Italy

**Keywords:** single-cell sequencing, myelodysplastic syndromes, acute myeloid leukemia, clonal hematopoiesis, immunotherapies, immune microenvironment

## Abstract

Myeloid neoplasms (MN) are heterogeneous clonal disorders arising from the expansion of hematopoietic stem and progenitor cells. In parallel with genetic and epigenetic dynamics, the immune system plays a critical role in modulating tumorigenesis, evolution and therapeutic resistance at the various stages of disease progression. Single-cell technologies represent powerful tools to assess the cellular composition of the complex tumor ecosystem and its immune environment, to dissect interactions between neoplastic and non-neoplastic components, and to decipher their functional heterogeneity and plasticity. In addition, recent progress in multi-omics approaches provide an unprecedented opportunity to study multiple molecular layers (DNA, RNA, proteins) at the level of single-cell or single cellular clones during disease evolution or in response to therapy. Applying single-cell technologies to MN holds the promise to uncover novel cell subsets or phenotypic states and highlight the connections between clonal evolution and immune escape, which is crucial to fully understand disease progression and therapeutic resistance. This review provides a perspective on the various opportunities and challenges in the field, focusing on key questions in MN research and discussing their translational value, particularly for the development of more efficient immunotherapies.

## Introduction

Myeloid neoplasms (MN) consist of a heterogeneous group of hematological cancers, arising from the hematopoietic stem cell (HSC) or progenitors in the bone marrow (BM) and sharing phenotypic features of the myeloid lineage (1). They include myeloproliferative neoplasms (MPN), which are featured by the hyperproliferation of near-normal maturing blood-cells; myelodysplastic syndromes (MDS), characterized by ineffective hematopoiesis, abnormalities in cell maturation and cytopenias; and acute myeloid leukemia (AML), which represents the most aggressive clinical phenotype, whose prominent features are the uncontrolled proliferation of immature hematopoietic precursors (i.e., blasts) and life-threatening BM failure (1).

The pathogenesis of MN is driven by the progressive selection of multiple genetic mutations (clonal evolution) (2–4). Somatic mutations can be identified in the peripheral blood of healthy subjects, a phenomenon known as clonal hematopoiesis (CH) that reflects the expansion of mutated HSC; by years or decades, CH may evolve to AML, eventually involving clinically recognizable pre-leukemic syndromes, such as MDS or MPN (5–10). In parallel, growing evidence also points to a prominent role of the immune system in shaping the evolution and clinical pictures of MN (11–13). The tumor immune microenvironment consists of multiple players, including adaptive and innate immune cells and stromal components, which may either antagonize or promote tumor progression; cancer cells themselves exhibit immunomodulatory properties and interact with microenvironmental components of the tumor niche (11–14). The connections between genetic evolution, changes in the immune microenvironment and clinical correlations, however, are poorly understood.

Single-cell sequencing technologies appear as ideal tools to investigate the highly-connected and plastic immune system. These technologies overcome the limited resolution of DNA and RNA sequencing of entire cell-populations (“bulk” sequencing), allowing deconvolution of heterogeneous populations and identification of rare cell types. Importantly, this is achieved by analyses of individual-cell transcriptional states, thus enabling the characterization of functional states while avoiding the bias of predefined lineage-markers (15, 16). Applications of single-cell technologies is continuously expanding with improving throughput, accuracy and reproducibility, thus making them widely adopted in cancer research, and being currently exploited for precision oncology (16, 17).

This review covers state-of-the-art single-cell technological applications with associated analysis methods; we aim to provide a perspective on the various opportunities to study the immune system and tumor microenvironment - for both experimental research and clinical translation - with a focus on specific issues relevant to MN.

## Current State-of-the-Art in Myeloid Neoplasms and Open Challenges

**Figure 1:**
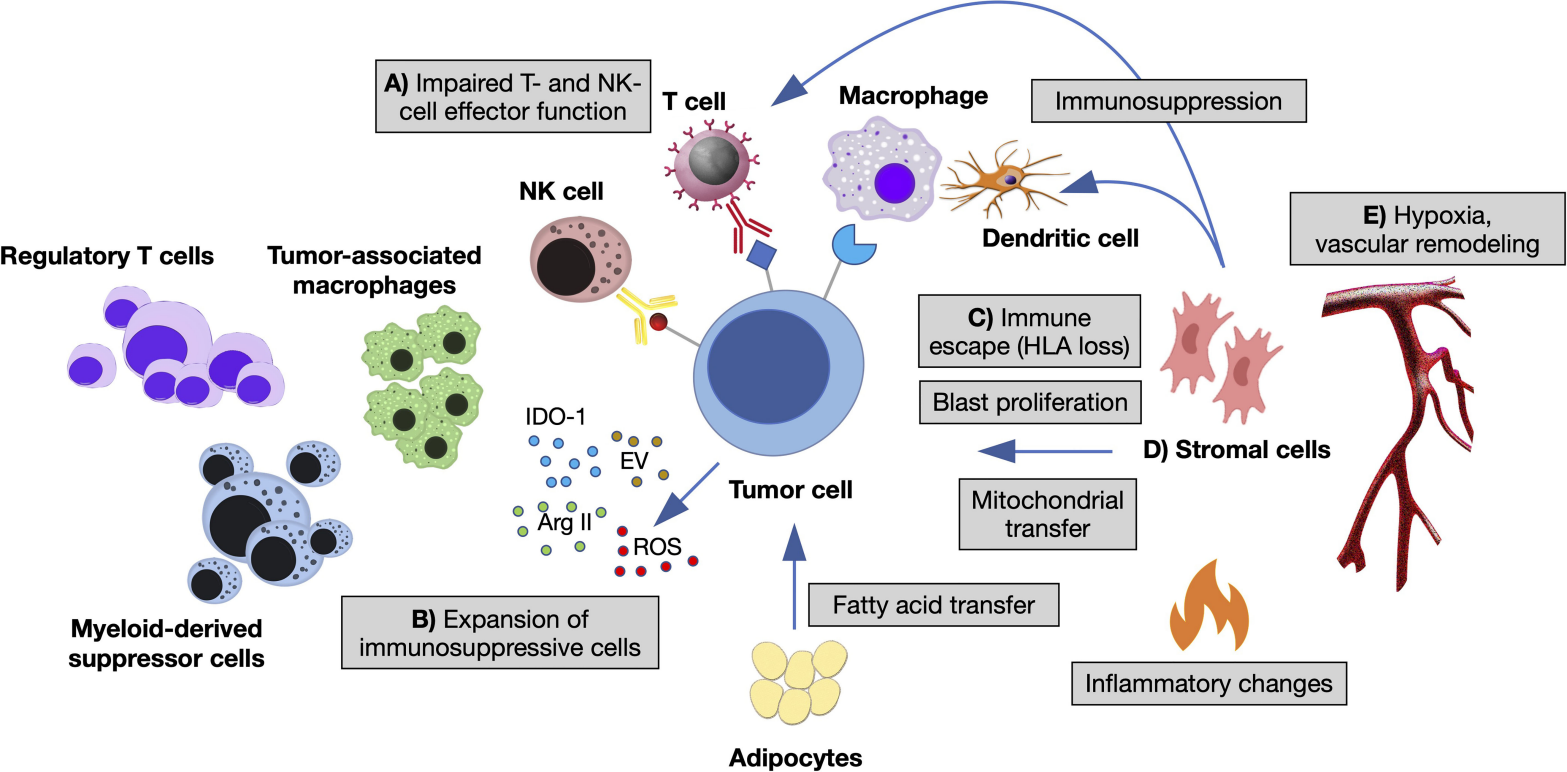
The immune microenvironment of myeloid neoplasms. Summary of the main interactions occurring between neoplastic cells and immune microenvironment in the bone marrow (BM) niche. **(A)** Impaired T- and NK-cell effector function by overexpression of inhibitory ligands (PD-L1, Gal-9, CD155, CD112, CD86, NKG2DL) and interaction with their respective receptors (TIGIT, TIM-3, PD1, CTLA-4, NKG2A); T-cell exhaustion and apoptosis driven by cytokine changes. **(B)** Expansion of immunosuppressive cells (regulatory T cells and myeloid-derived suppressor cells), switch of macrophages to tumor-associated macrophages by altered cytokine milieau and release within the BM niche of other soluble factors, such as reactive oxygen species (ROS), indoleamine 2,3- dioxygenase-1 (IDO1), arginase II (ArgII), and extracellular vesicles (EV). **(C)** Escape from macrophages and dendritic cells by decreased expression of antigen presentation molecules (HLA I and HLA II). **(D)** Stromal cells inhibiting the function of dendritic and T cells, influencing tumor proliferation and metabolic properties. **(E)** Vascular remodeling and hypoxia modifying immune cells’ homing and adhesion (11, 12, 14).

The immune microenvironment shapes MN through different branches of the immune system. A large body of pre-clinical and clinical studies indicate a key role of innate-immune cells and inflammation in the establishment of preleukemic states and their progression toward AML (13, 14, 18). For instance, the epigenetic reprogramming of aged HSC influences their response to inflammatory and immune-mediated signals, directly impacting on their division rate, myeloid-lineage skewing and survival advantage (5, 6). Adaptive immunity also plays a major role, as the presence of T cells at the tumor site is mandatory for recognition and elimination of transformed cells. Interestingly, its function changes according to the disease phase: in low-risk MDS, anti-leukemia cytotoxic (CD8+), helper (Th17) T cells and NK cells are expanded, in the presence of low counts of pro-leukemia T-regulatory lymphocytes (Treg); in high-risk MDS and AML, instead, Treg prevails over CD8+, Th17 and NK cells, suggesting that tumor clones acquire immune tolerance during disease progression (19, 20). In established AML, higher percentages of BM CD3+ and CD8+ T cells correlate with improved survival (21, 22) and response to the checkpoint inhibitor nivolumab (23). Importantly, expression of the checkpoint inhibitory receptor PD-1 and its ligand PD-L1 increases with disease progression of MN and AML relapse, as an immune-escape mechanism (24). Finally, leukemic blasts themselves modulate T- and NK-cell responses and are implicated in multiple mechanisms of immune evasion (25–28), in the context of an immunosuppressive microenvironment (**Figure 1**).

Prognosis and treatment of MN patients are extremely varying, depending on the disease entities and their associated clinical and molecular characteristics. Beyond achieving clinical control of hyperproliferation or cytopenias, the focus of management and research in MPN and MDS resides in predicting - possibly preventing - the evolution to AML. This is because, once leukemia is established, most AML patients ultimately succumb to their disease, despite some recent implementation of available treatments beyond the backbone of 7 + 3 chemotherapy. In fact, primary chemoresistance and relapse are the major causes of poor survival in high-risk MDS and AML patients (29–31).

The dynamics of leukemic progression, resistance to treatments and relapse have been mostly described in terms of genetic and epigenetic events, as associated to diverse synergistic combinations of mutations (2, 32–34); however, it is increasingly appreciated that genetic/epigenetic alterations do not entirely explain the complexity and heterogeneity of MN (35–37), as also inferred from the limited success of drugs targeting single genomic variants [e.g., FLT3 (38, 39) or IDH inhibitors (40, 41)] or epigenetic traits [e.g., hypomethylating agents, HMA (42, 43)]. Indeed, as featured above, there is increasing evidence of multiple mechanisms of immune-evasion during MN development; as a clinical correlate, the immunological eradication of therapy-resistant leukemia stem cells (LSC) by allogeneic hematopoietic stem cell transplant (alloHSCT) is the only strategy to overcome chemoresistance and obtain sustained remission (29, 44, 45). Therefore, a first major challenge in MN research is understanding the molecular mechanisms of immune-tolerance and the cellular relationships between the immune microenvironment and tumor clones during leukemic progression and therapeutic resistance; highlighting the connections between genetic evolution and immune escape seems particularly meaningful.

Although alloHSCT is an effective treatment, post-transplant relapse is a common occurrence, due to several leukemia-driven immune-escape mechanisms (12); also, its anti-tumor activity is rather poor in patients with active disease and, in general, it comes at the cost of high morbidity and mortality (46). These observations point toward the strong need of developing more potent, specific and possibly less toxic immunotherapeutic strategies for MN patients. These include harnessing T and NK-cell-mediated tumor clearance by checkpoint inhibitors, monoclonal antibodies, bispecific antibodies or chimeric antigen receptor (CAR) T cells; however, their effect has been less successful in MN than in other cancers (12, 47), despite the clear involvement of the immune system in MN pathogenesis. Reasons for this failure include a limited power of the currently used immunological markers to predict clinical response, the absence of a suitable target antigen and elusive resistance mechanisms. Thus, ongoing research efforts are committed to the discovery of druggable targets or mechanisms and more effective therapeutic combinations, which would benefit from a better understanding of the various cellular and functional components of the immune microenvironment.

## Overview of State-of-the-Art Single-Cell Technologies

### Limits of Bulk Sequencing and Promises of Single-Cell Technologies to Deconvolve the Immune Microenvironment of Myeloid Neoplasms

Traditional “bulk”-sequencing approaches rely on the analysis of whole samples through next-generation sequencing platforms, which generate multiple sequencing reads covering individual RNA or DNA molecules. Genomic and transcriptomic bulk data from the Cancer Genome Atlas (TCGA) Research Network have been crucial for our initial understanding of the tumor microenvironment and tumor-immune interactions. For example, Thorsson et al. (48) performed an extensive immunogenomic analysis of more than 10,000 tumors comprising 33 cancer types, and identified six immune subtypes that span cancer tissue and molecular subtypes, and differ by somatic aberrations, microenvironment, and survival; results from this study are available for exploration through the interactive Cancer Research Institute iAtlas portal (49). However, such resource is poorly applicable to MN research, as no MDS or MPN patients were included and limited data are available for AML, with no direct clues on tumor microenvironment composition, lymphocyte infiltration, immune features/modulators, immuno-oncology targets and associations with driver mutations (48). Furthermore, the output of bulk sequencing represents an “average” of the transcriptomic or genomic features of all sample cells, which poses a challenge in the precise deconvolution of intra-tumor heterogeneity. Dedicated bioinformatic tools have been developed to determine the composition of cancer microenvironments, including CIBERSORT, a method for estimating the relative proportions of cell types of interest in complex tissues from their gene expression profiles (50). However, this tool systematically over- or underestimates some cell types and requires a reference of gene expression signatures, which might bias the imputation of cells undergoing phenotypic plasticity or disease-induced dysregulation. Also, low intensity signals from rare cell populations might result undetectable with bulk sequencing approaches, which precludes the identification of rare (yet possibly functionally-relevant) cell populations. Therefore, deconvolution of the immune microenvironment can’t be comprehensively achieved from bulk studies.

Conversely, single-cell approaches allow the characterization of individual cells, thus providing a more faithful representation of the heterogeneity of tumor ecosystems (15, 51, 52). The use of single-cell technologies for research purposes is rapidly spreading, favored by combined academic and industrial efforts to improve standardization, develop several different applications and technological platforms and decrease costs. Some key aspects offer relevant advancement in the characterization of the immune tumor microenvironment: tumor and immune cells can be acquired in parallel without prior marker-based sorting, the high resolution of the approach allows the analysis of even small groups of cells with shared features, while the throughput of some sequencing platforms (up to thousands of cells) provides unprecedented statistical power. Moreover, cells can be investigated for both their phenotypic traits (e.g., surface markers, cell types) and functional states (e.g., over-expressed pathways, genomic features, activation of signalling pathways), which potentially opens perspectives on new mechanistic hypotheses (16, 52).

### Main Applications of Single-Cell Technologies

A comparative summary of single-cell methods for genomic studies is provided in **Table 1**.

- **Single-cell transcriptomics (scRNA-seq).** Recent advances in cell isolation methods and automated micro-fluidics techniques have improved tremendously the accuracy, sensitivity, reproducibility, and throughput of scRNA-seq, by which it is now possible to measure and model gene expression profiles from thousands of cells (64–67). A few scRNA-seq platforms are available on the market, differing by protocol complexity, costs, number of output cells, sequencing depth and full or partial coverage of transcripts (67). Such elements, as well as downstream analysis-pipelines, should be considered in view of the specific research-question. For instance, library construction methods that allow full transcript coverage (54, 68) are optimal for scoring expressed mutations, splicing isoforms (69) and T/B-cell receptor sequence (70–73), while molecular-counting methods based on the sequence the 5’ or 3’ end transcripts are better suited for cost-effective profiling of high numbers of cells and transcripts (64, 74). The introduction of unique molecular identifiers during library preparation allows counting and grouping of specific mRNA molecules prior to PCR amplification, thus increasing accuracy and reducing technical artifacts (75). High-dimensional scRNA-seq data need to be processed with specific computational algorithms, which incorporate various steps of quality control, normalization and dimensionality reduction to enable spatial representation (76–78). Opportunities from downstream analyses include unbiased clustering to identify groups of transcriptionally related cells, differential gene expression, and reconstructing dynamic biological processes, such as cellular differentiation and immune response, by inferring developmental ‘trajectories’ to reveal transitional states and cell fate decisions of distinct cell subpopulations (79–81).
- **Single-cell DNA sequencing (scDNA-seq).** scDNA-seq overcomes the limits of bulk sequencing allowing the direct identification of intratumoral genetic subclones - as defined by mutations co-occurring within the same cell - including rare clones, which may significantly impact tumor evolution and the acquisition of therapeutic resistance (3, 4, 82). The technique’s core involves whole-genome amplification (WGA) of single cells, which allows detection of single nucleotide variations, chromosomal copy number alterations or more complex genomic rearrangements. Droplet-based platforms currently enable high-throughput and cost-effective characterization of hundreds of amplicons in thousands of cells (59). However, a drawback of scDNA methods is the high rate of false negative and false positive hits, due to artifacts introduced during genomic amplification, non-uniform genome-coverage and allelic dropout events.
- **Single-cell epigenomics.** Bulk epigenomic techniques have been recently adapted to single-cell applications; analyses of chromatin organization and regulation enable to elucidate cell lineage and differentiation state in even thousands of individual cells simultaneously. Reported technologies allow scoring DNA-methylation patterns (by bisulfite-based sequencing such as scRRBS, scBS-seq, scWHBS) (60, 83, 84), chromatin regions available for transcription factors activity (scATAC-seq) (61), chromosomal conformations (scHi-C) (63) and histone modifications/binding sites (scChIC-seq) (85). However, these methods are limited by the low coverage of specific regulatory regions (such as enhancers).
- **Single-cell proteomics.** Though multiparameter flow-cytometry allows characterization of individual cells with multiple antibodies, the design of specific antibody-panels can be laborious and implicitly prevents unbiased and system-wide analyses. Predicting protein expression through scRNA-seq data, however, might be unreliable due the great extent of regulation of mRNAs and proteins at post-translational level. Recent technologies [such as CITE-seq (86) and REAP-seq (87)] partially overcame this limitation by combining oligonucleotide-labeled antibodies against cell surface proteins, thus enabling the simultaneous detection of gene expression patterns and protein levels in thousands of single cells in parallel. More than this, significant improvements were recently introduced into mass cytometry techniques (52). Mass cytometry uses antibodies labeled with heavy metals, whose presence and abundance are detected by a mass spectrometer; a key advantage over flow cytometry resides in the simultaneous detection of around 40 parameters per cell, for up to millions of cells, with significantly less spectral overlap. Thus, this high-dimensional assay enables a more thorough characterization and higher resolution of cellular sub-populations and individual cells, which can be especially useful when the total number of cells for evaluation is limited. Mass cytometry is particularly suitable for the study of tumor immune microenvironments, because it can characterize novel subpopulations of immune cell subsets and previously unrecognized aberrancies. Markers can be studied in combination, also through unsupervised clustering, and generate signatures that may incorporate relative abundance of different cell subsets, expression levels of different proteins, and/or activation states of various cellular signalling pathways. This can be exploited to discover biomarkers for disease classification and prognostication, and for predicting response to therapy. Mass cytometry also uniquely offer the important opportunity of unbiased identification of HLA-presented neoantigens (88), which are attractive target for immunotherapy as they are expected to drive highly specific and effective anti-cancer immune responses.

**Table 1:** Overview of selected single-cell technologies for genomic studies.

Method	Overview	Library construction	Features
***Single-cell transcriptome sequencing***			
Chromium (10× Genomics)	3’-end mRNA transcripts	GemCode (53)	Microdroplet-based method Advantages automatic cell isolation, cDNA synthesis, and amplification high number of cells (500-10,000/run) cell size up to 40 μm suitable to study individual cells in a large population use of UMIs mitigates amplification bias Drawbacks libraries of selected cells cannot be reanalyzed because libraries are mixed after barcoding diverse coverage across cells (5,000-10,00 reads/cell)
C1 Single-Cell Auto Prep system (Fluidigm)	Full-length cDNA	Smart-seq2 (54)	Microwell-based method Advantages automatic cell isolation, cDNA synthesis, and amplification can perform additional sequencing of libraries in user-selected wells stable coverage across cells (100-1,000 x 10^6^ reads/cell) suitable to study individual cells in detail Drawbacks limited number of cells (96-800/run) limited cell size (up to 25 μm) no UMI
	5′end mRNA transcripts	C1-CAGE (55)	
RamDA-seq (56)	Total RNA (full-length transcripts, long noncoding RNAs and enhancer RNAs)	RamDA retrotranscription	Advantages information on splicing events and enhancers possibility of automation with C1 Fluidigm platform Drawbacks no UMI high coverage requested high fraction of ribosomial RNA
***Single-cell genome sequencing***			
MDA (57)	SNVs	WGA; isothermal amplification	Advantages >99% genome coverage reduced representation bias as compared to PCR-based methods Drawbacks few tens of cells high rate of allelic dropouts
MALBAC (58)	SNVs, CNVs	preamplification + WGA	Advantages quasi-linear preamplification reduces WGA amplification bias 93% genome coverage 25x mean sequencing depth Drawbacks * few hundreds of cells
Tapestri (MissionBio) (59)	SNVs, CNVs on targeted loci	target amplification + barcoding for parallel processing	Microdroplet-based method uniform amplification across amplicons 20x mean sequencing depth thousands of cells suitable for clonal architecture reconstruction
***Single-cell epigenomics***			
scRRBS (60)	DNA methylation		hundreds/thousands of single-cells up to 1.5 million CpG sites (10% of genome) high rate of DNA degradation during bisulfite conversion
scATAC-seq (61)	Chromatin accessibility		possibility of automation with C1 and Chromium platforms thousands of cells
Drop-ChIP (62)	Histone modification		Hundreds of cells
Single-cell Hi-C (63)	Chromatin structure		Few tens of cells

UMI, unique molecular identifier; SNV, single-nucleotide variant; WGA, whole-genome amplification; MDA, multiple displacement amplification; MALBAC, multiple annealing and looping-based amplification cycles; CNV, copy-number variation; scRRBS, single-cell reduced representation bisulfite sequencing; scATAC, single-cell assay for transposase-accessible chromatin; ChIP, chromatin immunoprecipitation; Hi-C, high-throughput chromosome conformation capture.

### Challenges

Along with scientific opportunities, the adoption of single-cell technologies implies dealing with specific experimental and computational/statistical challenges, which are often shared across the different single-cell applications (89).

From the experimental point of view, the generation of single-cell data from a biological sample typically requires some common key steps (67, 89), including dissociation of cells from the tissue of interest, cell purification and isolation, library construction and sequencing. Each step impacts significantly the output results for downstream analyses. For instance, in scRNA-seq protocols, sample preparation and handling have to be carefully planned to avoid unnecessary stressful conditions, which are known to induce extensive cellular responses, thus introducing artifactual modifications of transcriptional states (90). The emergence of microfluidics techniques for cell isolation and combinatorial indexing strategies scaled up the number of cells being sequenced in one experiment and recently enabled multiplexing of different samples. Experimental steps, however, may result in considerable batch effect during later analysis and become the source of technical noise; this might be the case with protocols that use whole genome amplification, or the with carrying over of empty droplets during library preparation, cell doublets or dying cells.

In parallel, recurring computational challenges exist, due to inherent features of the sequencing data. The amount of material sequenced from single cells is considerably less than that available from bulk experiments, which leads to high levels of missing data. Missings may be due to technical dropouts (depending on platform and sequencing depth) or reflect true biological signal (as for variations in expression levels of a gene). This condition requires strategies to impute missing values, which have been more successful for genotype data than for transcriptomic data (89, 91). Conversely, any increase in the number of analyzed cells and features translates in the need of scalable data analysis models and methods. As a further complication, high-dimensional single-cell data have to be processed for easier tractability, while preserving the salient biological signals of the overall dataset.

Another common challenging task is the integration of multiple datasets for comparative analyses across multiple samples (even from different experiments or experimental conditions) (92–94). Computational approaches have been devised to score pairwise correspondences between single cells across datasets, enabling batch-effect correction and identification of populations with common sources of variation. This procedure, however, brings the inherent risk of overcorrection (95) and should be applied cautiously.

Finally, combining multiple types of information (such as DNA, RNA, proteins, epigenomics) on the same cell is crucial to get a more holistic view of cellular processes, but it requires the development of specific experimental settings and dedicated computational strategies to integrate complementary, possibly interdependent measurements. These approaches will be treated in a separate paragraph (see “*Integrating Complementary Cellular Information by Single-Cell Multi-Omics*”).

## Unravelling the Cellular Composition of Intra-Tumoral Healthy and Pathological Immune Microenvironments

The intra-tumoral immune microenvironment contains many different cell types, which exert their functions both independently and within cooperative networks (**Figure 1**). Hematopoietic cells include the adaptive (e.g., CD4+ and CD8+ T lymphocytes, B lymphocytes) and innate (e.g., NK lymphoid cells and macrophages) compartments of the immune system along with dendritic and myeloid-derived suppressor cells. Non-hematopoietic cells, instead, comprise mesenchymal stromal cells, adipocytes, osteoblasts, and cells from the vascular and neural niche. Malignant myeloid cells are themselves part of the immune microenvironment, as they crosstalk with other immune-competent cells (11, 13, 18). A better understanding of the immune tumor microenvironment requires the deconvolution of its cellular composition, which is preliminary for many secondary analyses.

The isolation of blood cancer cells for single-cell analysis is relatively simple as compared to solid tumors, since MN samples are most commonly collected as fresh mononuclear cells (MNC) isolated by Ficoll density gradient-centrifugation of the BM, thus not requiring tissue dissociation and preserving intra-tumoral hematopoietic cells. Non-hematopoietic cells, instead, are by far less abundant in the BM and peripheral blood and may require processing of larger samples (undigested BM or enzymatically digested bones) or specific purification steps, including depletion of the more abundant hematopoietic cells or positive selection using predefined lineage-markers (96). For these reasons, while these procedures have been used for the murine BM (97–99), the human counterpart currently remains uncharacterized.

### Innovation Given by Single-Cell Technologies

Single-cell technologies - mostly single-cell transcriptomics and proteomics - allow the analysis of both cell types (defined by phenotypic markers) and functional states, which spares the bias of using predefined lineage-markers (15, 16, 52) and opens innovative perspectives on our ability to classify the cellular components of immune processes. Indeed, emerging evidence from scRNA-based studies suggests that the physiological stages of hematopoietic differentiation, so far identified as discrete and homogenous subpopulations, are instead functionally heterogeneous and display lineage markers that overlap across different cell types, upon different biological conditions (100, 101). Also, the high resolution of single-cell transcriptional and proteomic data enables the recognition of intermediate or transitioning cell states, highlighting the continuity of biological processes. Given the plasticity of the immune system, these features are particularly attracting when applied to study the tumor microenvironment. The possibility of multimodal characterization, as obtained by quantifying both RNA and surface protein abundance (86), is especially promising for the discovery of previously unknown cell-subtypes and associated markers or gene-signatures (94).

### Challenges

Different computational approaches have been developed for the reconstruction and imputation of cell identities within both tumor and normal immune populations. A first, reference-free method consists in unsupervised clustering of scRNA-seq data followed by manual cell-type annotation according to cluster-level expression profiles (92, 102, 103). This approach, however, is time-consuming, limited in reproducibility and suffers from limited scalability to large datasets (104).

Specific cell-states might be more easily identified through a supervised analysis guided by an appropriate reference dataset. Such approach relies on mapping the query dataset onto an existing reference from pre-annotated and purified cell types, ideally characterized *via* the same technology (105). To this end, efforts to characterize the landscape of each human tissue and cell type at single-cell level are under the way, converging on the Human Cell Atlas project (106). Cell atlases are reference ‘coordinates’ that allow for the systematic mapping of cell types and states; for instance, comprehensive single-cell reference datasets are being developed for the human healthy BM and immune system, including both steady-state and perturbed conditions (107–113) (**Table 2**). The creation of a detailed “table of immune elements” including all immune types and states would be particularly useful to the purpose of classifying tumors according to immune subtypes, to make prognostic correlations and guide therapeutic assignment (16).

**Table 2:** Selected scRNA-seq datasets for the healthy and pathological human immune microenvironment.

Dataset	Tissue and cell populations	Condition	N cells/N individuals	Core features
Human Cell Atlas (107)	BM MNC	Healthy	103,000/8	Marker genes for cellular classification and trajectories Interactive web portal available
GSE120221, GSE120446 (108)	BM MNC	Healthy	76,645/20	Largest number of individuals Broad age range of donors Orthogonal validation by flow and mass cytometry Discrepancies in T and NK subsets
Human Cell Landscape (113)	BM MNC	Pathologic (cytopenias)	8,704/2	Atlas for cell-type identification Interactive web portal available Low sequencing depth
	PB MNC	Healthy	17,331/4	
TMExplorer (114)	BM MNC	Pathologic [AML (102), CML (115)]	AML: 38,410/40CML: 2,287/20	Collection of microenvironment datasets from 12 different cancer types R package interface to access datasets and metadata Provides gene expression data, cell type annotations and gene-signature information
GSE126030 (110)	T cells (lungs, lymph nodes, BM and PB)	Healthy (resting and activated)	50,000/4	Reference map of human T cells functions related to tissue site vs PB Applied to score distinct tumor-associated phenotypes

BM, bone marrow; MNC, mononuclear cells; AML, acute myeloid leukemia; CML, chronic myelogenous leukemia; PB, peripheral blood.

However, this is currently hard to achieve, due to the inherent plasticity of the immune system and the high variability between individuals. Also, tumor and microenvironment cells may show several and dynamically changing aberrancies, as compared to the healthy tissue counterpart. This poses a limit to our ability to recognize rigidly distinct cell types. In fact, as less-characterized disease entities and large patient cohorts are being studied, many yet-uncharacterized immune cells and pathways will emerge, further challenging current models of immune identity. Analytical pipelines should account for the uncertainty of mapping unknown cell type/state; for instance, a recently published tool for cell-type annotation (CellAssign) (116) leverages prior knowledge of lineage-specific marker genes to annotate scRNA-seq data into predefined or novel cell types, based on a probabilistic model. Orthogonal validation with flow and mass cytometry data, as well as integrating transcriptional data with protein expression and scDNA-seq, are expected to further refine current single-cell classifications.

### Application in the Current MN Research

The ability to assign cell identity in hematopoietic tissues has been validated for both scRNA-seq and mass spectrometry, although the latter is more precise in distinguishing immune cells with closely overlapping transcriptional profiles, such as T and NK cell subsets (108). In a recent work, such a technology has been used to classify subsets of NK cells in 48 newly diagnosed AML and 18 healthy subjects (117). AML samples showed an accumulation of aberrant CD56−CD16+ NK cells, which was associated with an adverse clinical outcome and decreased overall survival. High-dimensional characterization of this NK subset highlighted a decreased expression of some receptors required for antileukemic activation, such as NKG2D, DNAM-1, and CD96; the Authors concluded that the accumulation of CD56−CD16+ NK cells, combined with the reduced frequency of conventional NK subtypes, may be the consequence of escape from innate immunity during AML progression. Subsets of monocytes were found to be decreased in MDS BM, which mediated the expansion of a specific T cell pool (118). Mass spectrometry also enables recognizing aberrant myeloid differentiation patterns, as recently demonstrated on MDS samples compared to healthy donors (119).

Importantly, dissecting the cellular composition of the immune microenvironment can be applied to highlight changes across disease and/or treatment phases. For instance, the seminal study from van Galen et al. (120) employed scRNA-seq to characterize BM MNC from 16 AML patients at diagnosis and during treatment. Results showed great variations in the proportions of cell types during the clinical course, consistently with immunohistochemistry; AML BM generally presented with fewer cytotoxic T cells than healthy donors, yet greater numbers of Tregs, which confirmed previous findings and established the existence of an immunosuppressive tumor microenvironment in AML. Further mechanistic studies are needed to link changes in immune subsets or immune targets to dynamics of relapse. As LSC are deemed to be responsible for AML relapse, their identification and characterization is particularly critical for the development of efficient immunotherapies. In this regard, Levine et al. published PhenoGraph, a software for analyzing mass cytometry data that enabled better identification and characterization of LSC (102). Moreover, one recent paper used mass cytometry and RNA-seq to feature CD200 as a LSC–specific immune checkpoint overexpressed in AML LSC (121).

## Discovering Functional Phenotypes, Molecular Mechanisms and Biomarkers

Immune responses are plastic and can be extremely heterogenous, depending on tissues, environmental contexts, healthy or pathological conditions (122–124). Commonly-used small sets of markers fail to describe the full spectrum of functional states and inherent gene expression programs, which, instead, can be optimally captured by high-dimensional single-cell analyses (16, 125, 126). In contrast to marker-based methodologies that seek for rigid separation of defined entities, single-cell technologies allow to set broadly inclusive experiments without *a priori* marker selection, enabling data-driven analyses on all cell populations involved in a given condition. The first large-scale ‘ecosystem-wide’ scRNA-seq study was performed by Tirosh et al. on melanoma patients (127). In the context of MN research, Van Galen’s paper is a paradigmatic example for this approach.

In order to define functional subsets among AML-associated immune cells, Guo et al. (128) re-analyzed the scRNA-seq dataset from the aforementioned study (120), focusing on non-blasts AML cells and 4 healthy BM donors. The study concluded that AML coexists with highly heterogeneous immune effectors and suppressive subsets, which showed common features of functional aberrancy and exhaustion of possible prognostic significance. To the same aim, one group developed an integrated functional approach coupling mass cytometry coupled to cytokines profiles (129) and applied it to 49 AML patients, confirming functional impairment of AML-associated T cells mediated by immune checkpoints (130). Single-cell transcriptomic has been applied in both animal models (131) and cancer patients (132, 133) to investigate changes in the tumor microenvironment upon treatment with immune checkpoint inhibitors, to the end of finding response-associated signatures. Following the same approach, one small study used mass cytometry on serially collected samples from 9 AML patients treated with HMA and avelumab, a PD-L1 inhibitor; the ratio of CD4/CD8 and composition of residual T cells emerged as the most important predictors of response to treatment, and AML cells expressed a variety of other immune checkpoints (such as PD-L2, OX40, TIM3) that might be considered for future combination therapy (134).

Regarding the direct role of malignant cells in shaping the immune microenvironment, van Galen et al. found that AML cells exhibited marked intra-tumoral heterogeneity, with “primitive-like” cell-types showing dysregulated co-expression of stemness and myeloid commitment genes, and more differentiated “monocyte-like” cell-types showing immunomodulatory properties linked to T-cell suppression (120). These two different cell states were obtained by classifying malignant cells according to their similarity to normal hematopoietic cell types and resulted associated to specific gene signatures. Specifically, the direct comparison of leukemic versus normal cells revealed 296 genes that were preferentially expressed in malignant monocyte-like cells from one or more AML samples, including genes associated with myeloid-derived suppressor cells, antigen presentation components and leukocyte immunoglobulin-like receptors, such as tumor necrosis factor and interleukin-10 pathway genes or regulators of reactive oxygen species. Although expression of these genes markedly varied among patients, most samples expressed high levels of CD206/MRC1 and CD163, two surface markers associated with immunosuppressive myeloid cells (132), whose expression was also found to be associated with poor outcome in the TCGA AML-cohort (135). Thus, though highly heterogenous, the different expression programs identified by scRNA-seq might converge on common functional pathways of prognostic and therapeutic interest. Tightly correlated gene modules can reveal how specific pathways and cellular functions (e.g., proliferation, antigen presentation, exhaustion, differentiation, etc.) are distributed across cell types, thus defining specific immunomodulatory patterns. Thereafter, detailed analyses can be restricted to cells expressing common transcriptional modules, an approach that may lead to the identification of new surface markers, immunoregulatory molecules or tumor-specific antigens for therapeutic exploitation. A catalogue of AML-specific antigens and corresponding HLA ligands has been previously obtained by mass spectrometry characterization (88).

Additional molecular mechanisms for tumor-related immune changes include epigenetic dysregulation, which may affect T cell differentiation and functions by remodeling active-enhancer landscape and transcription factor binding (136–141). One notable example is the documented increased chromatin accessibility at the enhancer site of PDCD1, the gene encoding the checkpoint inhibitor PD-1 (142). A proper T cell functionality is needed to convey the effect of many immunotherapeutics; in this context, a recent study applied scATAC-seq to characterize chromatin profiles of ~200,000 single cells in both peripheral blood and basal cell carcinoma samples before and after PD-1 blockade therapy, which identified chromatin regulators of therapy-responsive T cell subsets at the level of individual genes and regulatory DNA elements (143). This is a critical field of investigation in MN research, since studies have shown that during disease progression the adaptive immune microenvironment switches from cytotoxic to regulatory, suggesting the appearance of immune tolerance (19, 20) and immune-escape mechanism (24); also, T cell exhaustion has been recognized as a cause of failure of autologous CARTs (136).

## Shaping the Immune Microenvironment by Cell-to-Cell Interactions

In either the physiological or tumor microenvironments, immune cells should not be considered as functionally separate entities, as immune processes are mediated by networks of tissue-resident and/or circulating cell types. These interactions respond dynamically to environmental stimuli, possibly driving disease progression and sensitivity or resistance to immunotherapies (137). Thus, identifying critical signalling pathways underlying the network of immune-cell interactions is critical to predict cancer phenotypes, identify druggable genes or manipulate the immune system for therapeutic purposes (138) (for example, by genome editing (139) and cell engineering to control how pairs of cells interact). A critical starting point is the analysis of the coordinated expression of known ligands and their cognate receptors across different cell types. This can be achieved, for example, by combining information from protein–protein interaction databases (140, 141, 144, 145) and single-cell technologies. Alternative strategies for deciphering cell–cell interactions incorporate downstream signalling, gene regulatory networks and metabolite secretion coupled with advanced statistical methods [reviewed by Armingol et al. (137)].

A further application of single-cell transcriptomic is represented by the integration of transcriptomic profiles of single cells with their spatial position in tissue contexts, an approach that allows mapping tumor cells with respect to other cell types or relevant tumor areas, such as vessels or the tumor edge, and that can be used as a guide for refining cell type identification, monitoring cell abundance, behavior and interactions upon different disease or treatment phases (146–148). Various technologies and computational tools exist to profile hundreds to thousands of transcripts at different resolutions, which have been mostly applied to generate spatial transcriptomic maps of solid tumors (149–157). Of note in the context of MN, Baccin et al. developed LCM-seq, a laser-capture microdissection and sequencing protocol specifically designed to capture the three-dimensional organization of BM cell populations and their location within distinct niches (98). Alternative approaches are based on the recovery of specific neighboring cells, as in the PIC-seq (158) and NICHE-seq (159). In the PIC-seq, tissues are mildly dissociated to retain *in situ* cellular structures, physically interacting cells (PICs) are then recovered by FACS-sorting using specific markers and subjected to scRNAseq (158). In the NICHE-seq, instead, cells interacting with specialized niches within organs are identified in model systems using photoactivatable fluorescent reporters (159).

Finally, alternative single-cell technologies are emerging to overcome the limits of RNA analyses, e.g. the lack of information on post-transcriptional and post-translational processing (160). Spatial resolution, in fact, can also be achieved by immunohistochemistry coupled to mass spectrometry, a technology that allows the detection of up to 40 proteins with a subcellular resolution of 1μm (161, 162). One recent study, as an example, applied multispectral imaging to understand the spatial relationship between CD34+ hematopoietic cells and immune cell subpopulations in the BM of MDS and secondary AML samples. CD8+ and FOXP3+ T cells were regularly seen in close proximity of CD34+ MDS/AML, yet not in controls; this finding correlated to blast counts but not to genetics, and the frequencies of immune cell subsets also differed in MDS and sAML when compared to controls, providing novel insights in the dynamics of immune deregulation during MN evolution (163). Methods that allow an accurate view of intercellular communication include Nativeomics (164), which detects intact ligand–receptor assemblies using mass spectrometry, single-cell proteomics (165) and INs-seq (166), which couple scRNA-seq with intracellular protein measurements to simultaneously profile transcription factors, signalling activity and metabolism. In all cases, mechanistic hypotheses generated by computational inferences of single-cell data should undergo careful validation using orthogonal technologies, including confirmation of the expression of candidate proteins (e.g., with proteomics, enzyme-linked immunosorbent assay, western blot or immunohistochemistry), or direct visualization of interacting cells.

## Integrating Complementary Cellular Information by Single-Cell Multi-Omics

Several emerging single-cell technologies are committed to recording complementary types of cellular and molecular information from the same cell, including its transcriptome, genome, epigenome, proteome and spatial localization (**Table 3**). The application of multi-omics approaches enables the integration of different molecular layers within single cells at the same time and, possibly, with respect to their surrounding environment, thus providing an unprecedent description of the cancer ecosystem.

**Table 3:** Overview of selected single-cell multi-omics methods.

Method	Overview	Throughput (N cells with multiomics characterization)	Features	Limits
***Genome + Transcriptome***				
G&T-seq (167)	Experimental methodPhysical separation of RNA and DNA with subsequent parallel amplification and sequencing	-/+	CNV (direct scoring) SNV (direct scoring) Full-length transcriptome (including fusions)	Low throughput Low coverage
HoneyBADGER (168)	Computational methodIntegration of normalized scRNA-seq profiles as compared to:- putative diploid reference of comparable cell type- allelic frequency of heterozygous germline SNP		CNV (inferred from scRNA-seq) LOH (inferred from scRNA-seq) Transcriptome	No information on DNA alterations smaller than 10 megabases Best performance with scRNA-seq protocols that achieve full-transcript coverage
Scmut (115)	Computational methodVariant calling implemented to both scRNA-seq and WES data		Expressed SNV (inferred from scRNA-seq) Transcriptome	Relies on quality of the alignment and transcript annotation Detection sensitivity of a mutation depends on the corresponding gene expression High rate of false positives and negatives
Van Galen et al. (120)	Experimental methodTarget amplification of transcript and locus of interest, integration with long-read sequencing	+	Expressed SNV (inferred from scRNA-seq), insertions, deletions and fusions Transcriptome	Depends on expression for mutation detection
Petti et al. (169)	Experimental methodVariants scored in WGS and then detected in scRNA-seq data	++	Expressed SNV (inferred from scRNA-seq), indels Transcriptome High-throughput that preserves biological complexity General applicability	5’-end bias Heavily depends on expression for mutation detection No clonal reconstruction (wild-type status not defined)
GoT (170)	Experimental methodTarget amplification and circularization of transcript and locus of interest	++	Expressed SNV (inferred from scRNA-seq) Transcriptome Overcomes end bias by transcripts circularization	Depends on expression for mutation detection (mitigated by target amplification)
TARGET-seq (171)	Experimental methodRelease of gDNA and mRNA followed by target amplification	+	SNV, indels Transcriptome Parallel information from coding and non-coding DNA Clonal reconstruction Low allelic dropout	End-bias with ‘high-throughput’ protocol
***Genome + Proteins***				
Tapestri (Mission Bio, Inc) (3, 59)	Experimental methodMicrofluidic workflow for target amplification of DNA amplicons and proteins	++	SNV CNV Cell-surface proteins Standardized commercial platform Customizable gene and antibody panel Clonal reconstruction at single-cell level Integrated pipeline for multi-omics analysis	No information on gene expression and regulatory networks
***Transcriptome + Epigenome***				
scM&Tseq (172)	Experimental methodPhysical separation of RNA and DNA, which allows for bisulfite conversion of DNA without affecting the transcriptome	-/+	Transcriptome Methylome	Low sequencing depth Low throughput
Paired-seq (173)	Experimental methodLigation-based tagging of both open chromatin fragments and cDNA	+++	Transcriptome Chromatin accessibility Extremely high throughput (up to millions of cells)	Non optimal library complexity
***Transcriptome + Proteins***				
CITE-seq (86)	Experimental methodAntibody-bound oligos act as synthetic transcripts that are captured during most large-scale oligodT-based scRNA-seq library preparation protocols	++	Transcriptome Surface proteins Adaptable to RNA interference assays, CRISPR, and other gene editing techniques. No upper limit in number of antibodies	No spatial information No intracellular proteins
PLAYR (174)	Experimental methodLabelling of RNA and proteins with isotope-conjugated probes andantibodies for mass spectrometry detection	+	Transcriptome Surface and intracellular proteins	No spatial information- Limited number of proteins
***Transcriptome + T cell receptor***				
Tessa (111)	Computational methodBayesian model trained on bulk and scRNA-seq of TCR and T cells		TCR sequences Transcriptome	No information on splicing isoforms
RAGE-seq (73)	Experimental methodCombined targeted capture and long-read sequencing of full-length transcripts	++	TCR/BCR sequences Transcriptome Splicing isoforms Accurate antigen receptor sequences at nucleotide resolution Information on splicing isoforms Adaptable to any scRNA-seq platform using 3′ or 5′ cell-barcode tagging	Low recovery of cell barcodes due to low accuracy of long-read sequencing Possible PCR artifacts

CNV, copy number variation; LOH, loss of heterozygosity; SNP, single nucleotide polymorphism; SNV, single nucleotide variant; WES, whole exome sequencing; WGS, whole genome sequencing; gDNA, genomic DNA; cDNA, coding DNA; PCR, polymerase chain reaction; TCR, T cell receptor; BCR, B cell receptor.

-/+, tens of cells; +, tens of cells; ++, hundreds of cells; +++, thousands of cells.

### Genomic Data Combined With Transcriptome/Proteins

Because of the prominent role of genetics in cancer biology and clinical management, most efforts have converged on the development of technologies that jointly capture a single cell’s genomic profile along with its phenotypes defined by either surface markers or functional features. A number of strategies have been published, each with its own strengths and limits (**Table 2**), which hold enormous potential for the study of the immune microenvironment in MN. Direct approaches analyzing genomic DNA along with mRNA are technically limited by the low DNA sequencing coverage that can be achieved at single-cell level, and are consequently hampered in their sensitivity (167, 175). This limit can be circumvented using indirect approaches, which aim at identifying expressed genomic variants in scRNA-seq data and allow the analysis of high numbers of cells, thus preserving the biological heterogeneity of the sample (115, 120, 169–171, 176, 177). Experimental and computational methods are under continuous development to achieve the broadest applicability. Another approach was featured in the seminal paper by Miles et al. and consists in combining scDNA-seq with cell-surface protein expression, which the Authors exploited to characterize CH, MPN and AML patients (3).

A first application of combined genomic/phenotypic approaches is the distinction of neoplastic from non-neoplastic cells within tumors, which remains inaccurate when solely based on the expression of specific genes or surface markers, due to the occurrence of technical artifacts in scRNA-seq or aberrant expression in either cell-populations. Mapping single-nucleotide variants and/or copy-number variations across phenotypically defined cells can enhance the confidence of such imputation (51). In principle, the acquisition of thousands of unselected cells (e.g., total CD34+ or BM/PB MNCs) would allow the characterization of both neoplastic and non-neoplastic/immune compartments in parallel, to study the functional properties specific to each compartment and clone. Only a few studies have exploited such approaches in MN, focusing on the mapping of single mutations. Giustacchini et al. obtained scRNA-seq profiling of BCR-ABL positive vs negative HSC from patients with chronic myeloid leukemia, and found restricted expression in BCR-ABL negative HSC of inflammatory genes with suppressor functions on HSC (i.e., IL6 and its downstream mediators, TGF-β and TNF-α pathways) (176). Another study used transcriptional and mutational single-cell data to feed a machine-learning model for the identification of malignant vs non-malignant AML cells, and found heterogenous malignant cell-types whose abundance correlated with genotypes and survival (120). Indeed, future studies employing multi-omics single-cell strategies will be instrumental to detail the molecular mechanisms by which tumor cells harboring specific genomic alterations interact with their own immune microenvironment, potentially driving immune escape and response to immune-therapies. Preliminary evidence supports this perspective with different mechanisms, such as the expansion of specific immune populations [e.g. in MDS, where chromosome 8 trisomy and consequent WT1 overexpression fuel CD8+ expansion (178)]; the up/downregulation of immune effectors activity [e.g., fusion proteins PML-RARα and AML1-ETO impair NK cytolytic activity by downregulating their receptor’s ligand CD48 on AML cells (179)]; enhancement of specific signalling and immune activation pathways [such as for mutations in JAK2 (180–182) or spliceosome genes (12, 183), which are early genetic events in MN, or for signalling effector mutations, which occur in late AML subclones (3)].

Immunomodulation by either tumor or micro-environment cells has been recognized as a further mechanism that influences the dynamics of clonal expansion in MN. Dysregulation of innate immune and inflammatory cells and signalling contributes to the competitive advantage of CH-mutant HSC during aging, particularly in the context of TET2, DNMT3A and JAK2 mutations (5, 6, 182, 184–186). In addition, mutations associated with CH are nearly always present in circulating innate immune cells and, less frequently, in the T and B lymphoid compartment, which might affect immune surveillance against emerging tumor cells and response to immune therapies (187). Understanding the molecular and cellular relationships between the immune microenvironment and preleukemic clones remains a crucial step to efficiently track - and possibly intercept - the evolution to AML, as the risk of leukemic transformation varies significantly across CH-individuals and pre-leukemic patients and is associated to diverse synergistic combinations of mutations. Although not specifically focusing on the immune microenvironment, Miles et al. observed differential skew to the myeloid, B or T cell lineages, depending on which CH gene was mutated; genotype-driven changes in cell-surface protein expression were also reported in the leukemic phase, with signaling effector mutations leading to increased CD11b expression (3). In established AML, the same information might instead aid in understanding the molecular basis of chemoresistance and the jeopardized response to various immunotherapeutic strategies. In this context, common AML-associated translocations (AML1-ETO, DEC-CAN, PML-RARα, BCR-ABL) or mutations (FLT3-ITD, NPM1, IDH1^R132H^, mutations in spliceosome genes and some TP53 hotspots, JAK2, CALR) produce MN-specific immunogenic proteins that may become ideal antigen targets for the development of immunotherapies (12, 188).

### Transcriptomic Data Combined With T Cell Receptor Information

Finally, the T- or B-cell receptor repertoire of individual lymphocytes can be scored in parallel with their gene expression profiles, using properly devised experimental and computational methods on scRNA-seq data (73, 111), thus providing connections between lymphocyte clonality and functional responses, which can inform the discovery of antigen-reactive antibody candidates, antigen targeting efficiency of T cell clonotypes, and evolution and response to various immunotherapies.

### Transcriptomic Data Combined With Proteomic Data

Technologies are also available that allow concomitant analyses of protein and transcripts at single-cell levels. They are particularly useful to investigate post-translational regulatory events and to relate functionally-defined phenotypes to protein markers, which might assist tumor classification, biomarker assessment for prognostic purposes, and development of therapeutic targets. Surface proteins can be detected by implementing gene-expression libraries with oligonucleotide-labeled antibodies, as for the above-mentioned CITE-seq (94) and REAP-seq (87). Notably, the CITE-seq workflow is compatible with the most frequently used commercial platforms for scRNA-seq, and there’s no upper limit to the number of antibodies that can be used. PLAYR, instead, relies on mass spectrometry and allows the detection of up to 40 proteins (174). This technique might be critical when high-quality antibodies are unavailable; also, it can be deployed for index sorting and imaging approaches to enable spatial resolution. Using other techniques, intracellular proteins can be accessed as well with scaling throughput (189, 190).

### Transcriptomic Data Combined With Epigenomic Data

Various single-cell technologies are becoming available for the simultaneous analyses of expression, DNA methylation or chromatin accessibility (**Table 2**). This level of investigation would be particularly important to characterize MN, as epigenomic changes occurring in either tumor or immune cells are relevant to aberrant hematopoietic differentiation (191), genetic-independent disease progression (32) and immune functions (132, 142, 192–194). Moreover, HMA [which are typically used in older MDS or AML patients (29)] have been found to potentiate the immunogenicity and the immune recognition of neoplastic cells by up-regulating the expression of molecules that are crucial in host-tumor immune interactions (195–197), which makes them an ideal partner for combination with immunotherapeutic agents (23).

### Triple-Omics

Finally, although preliminary, recent studies have reported the development of single-cell triple-omics sequencing techniques, such as for the joint capture of the transcriptome, genome and DNA methylome [scTrio-seq (198)]; transcription, DNA methylation and chromatin accessibility [scNMT-seq (199)]; or transcription, chromatin accessibility and surface proteins (200).

## Open Perspectives and Future Directions

**Figure 2:**
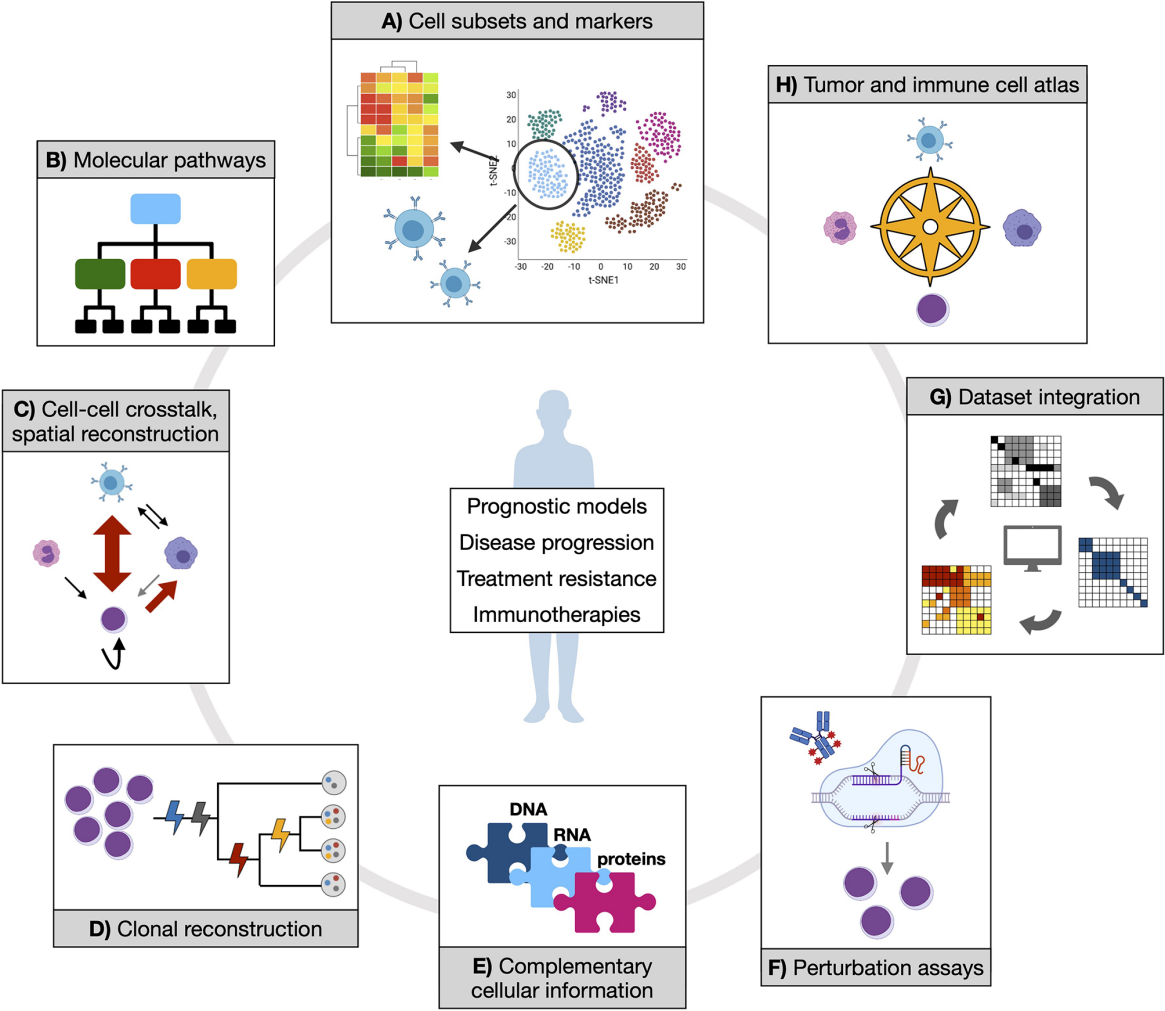
Opportunities of applying single-cell technologies to characterize myeloid neoplasms. Established and novel single-cell technologies can provide manifold information to address clinically relevant questions and contribute to therapy development. **(A)** Isolating cell subsets from transcriptional data could score functional populations (whose markers can be defined in the same context by either gene expression or proteomic data) that might be associated to prognostic features or treatment response. Also, T or B cell receptor clonality can be studied in parallel with associated transcriptome, which would shed light on expansion dynamics of T and B populations in physiology and tumor or upon treatment. **(B)** Inferring molecular pathways (at gene expression or epigenetic level) from such populations might reveal distinct or convergent functional modules, potentially simplifying disease heterogeneity with implications for therapeutic exploitation. **(C)** Cell-cell crosstalk and spatial reconstruction by transcriptomics are fundamental notions to score cancer and immune cells interactions in their proper environmental context, enabling more precise mechanistic and regulatory insights. **(D)** Clonal reconstruction is one core objective of single-cell DNA analysis in myeloid neoplasms, and a mainstay to understand (and potentially prevent) disease evolution. **(E)** Different coexisting molecular layers can be complemented, experimentally and/or computationally, to uncover previously hidden information and mechanistic hypotheses. **(F)** Perturbation assays offer experimental ways to tackle specific functional processes (such as drug response), which can be further dissected by coupling experimental read-out with omics. **(G)** Integration of different datasets are expected to increase statistical power and accuracy of previous observations. **(H)** All of the generated knowledge might enable the creation of an atlas for tumor and immune cell types and states, which would represent a comprehensive reference resource for future studies.

Despite the number of single-cell approaches that have been developed in the last few years, and the fewer proof-of-concept applications, most of the relevant questions in the field of MN remain to be addressed (**Figure 2**).

So far, single-cell studies aiming to describe the immune microenvironment in MN have mainly focused on AML, while a thorough characterization of CH, MDS and MPN samples is currently lacking. To formally address questions about disease progression, therapeutic resistance and relapse, more informative research should be performed by prospective monitoring of MN evolution. Following the history of MN patients at multiple time-points should allow tracing of evolving cellular clones across different disease stages, as well as residual disease [scored by immunophenotypic markers, genetic markers or both (6)] after treatment and donor chimerism after alloHSCT, in the context of surrounding immune cells. It is envisioned that such prospective biobanks for single-cell characterization might uncover immune-related pathways that can be targeted for reducing the selective advantage of the CH or MDS transforming clones, an approach supported by proof-of-concept studies in murine models (185, 186). Also, the same strategy could detail the molecular mechanisms of resistance and immune evasion and monitor variability in treatment response. Finally, given the association of immunomodulatory features with both disease progression and survival, there is also a rationale for studying the inclusion of immunologic parameters to refine prognostic models currently used for MDS (201, 202) and AML (22, 29, 203) patients.

Resistance to treatment (including chemotherapy or HMA, target therapies and immunotherapies) represents the main cause for poor survival in AML, which is the final stage of the MN’s natural history (29, 30, 46). Resistance and relapse involve genetic and epigenetic dynamics of cell clones in parallel with changes in the immunomodulatory properties of both tumor and immune cells (204–207), whose interplay can be best understood by single-cell multi-omics approaches. Novel single-cell approaches to tackle therapy-resistant cells in model systems include the use of expressed barcodes, which enable the simultaneous recording of clonal evolution and transcriptional phenotypes, eventually coupled to genetic perturbations (74, 208, 209), to study mechanisms of immune evasion. Similarly, other methods can score specific cell clones (including HSC, preleukemic and leukemic stem cells) *via* lineage barcoding and tracing (101, 210, 211), while pulse-chase, inducible lineage tracing methodologies can record past events, such as cell divisions, enabling analyses of cell cycle properties (212, 213). LSC are more frequently quiescent (i.e., not proliferating) than normal HSC, a state that may mediate chemoresistance and relapse; regulation of quiescence can be driven by cell-autonomous genetic or epigenetic changes, but also interactions with the BM immune microenvironment (11, 214, 215), which provides another important hint for clinical translation.

Finally, a further major challenge in MN-related research is the development of effective immunotherapeutic approaches. As discussed above, scRNA-seq and mass cytometry have the capability to identify cell populations with specific functional properties in both tumor and immune compartments. Describing associated molecular markers might aid the process of selecting target antigens in the design of immunotherapies, especially when scRNA-seq is coupled to surface proteins detection in CITE-seq (86) or other platforms for the analyses of cell-to-cell and spatial interactions (137). Since MN are not featured by a single and common surface-antigen with druggable characteristics, as it is, for example, CD19 in B lymphoblastic leukemia (216), multi-omics represent promising strategies to identify different combinations of candidate targets and/or involved pathways.

With the advancement of innovative methodologies, the number and scale of publicly available datasets are continuously increasing (217–220); this offers the opportunity to integrate and interrogate multiple datasets for the validation of previous discoveries or, conversely, the generation of new hypotheses to be experimentally validated, and will possibly allow the construction of a specific cell-type atlas for both cancer and immune cells. Proper curation, quality control and reliable computational strategies for integration are essential to the full exploitation of available data. However, comprehensive integration is challenging because datasets are typically generated through a variety of different approaches and heterogeneous study designs (95). To this aim, achieving standardization of experimental protocols will play an important role. Ongoing and future efforts are committed to identify and benchmark optimal computational methods for data integration, and to improve data sharing and accessibility (221).

## Conclusions

In conclusion, although very few data exist specific to MN, single-cell technologies - especially those providing multi-omic measurements of the same single cell - hold the promise to yield comprehensive insights into how pre-leukemic and leukemic cells interact with the different players of the associated immune microenvironment. The spreading availability and scaling of the various single-cell approaches is expected to enable the characterization of large clinical cohorts involving patients with different MN types, upon different treatment conditions, as well as more focused experimental models. Despite many challenges to solve, these efforts will build a detailed ecosystem-level picture of MN to help highlight new hypotheses and research directions, inform dynamics of progression, select targeted drugs and rational combinations, and predict efficacy of immunotherapy.

## Author Contributions

CC and PGP designed the research. All authors contributed to writing and reviewing the manuscript. All authors approved the final version of the manuscript.

## Funding

This work was supported by AIRC IG 2017 - 20162.

## Conflict of Interest

The authors declare that the research was conducted in the absence of any commercial or financial relationships that could be construed as a potential conflict of interest.

## Publisher’s Note

All claims expressed in this article are solely those of the authors and do not necessarily represent those of their affiliated organizations, or those of the publisher, the editors and the reviewers. Any product that may be evaluated in this article, or claim that may be made by its manufacturer, is not guaranteed or endorsed by the publisher.
