# Corrigendum: Single-cell technologies to decipher the immune microenvironment in myeloid neoplasms: Perspectives and opportunities

**DOI:** 10.3389/fonc.2022.974476

**Published:** 2022-08-15

**Authors:** Chiara Caprioli, Iman Nazari, Sara Milovanovic, Pier Giuseppe Pelicci

**Affiliations:** ^1^ Department of Experimental Oncology, IRCCS Istituto Europeo di Oncologia, Milan, Italy; ^2^ Scuola Europea di Medicina Molecolare (SEMM) European School of Molecular Medicine, Milan, Italy; ^3^ Hematology and Bone Marrow Transplant Unit, Papa Giovanni XXIII Hospital, Bergamo, Italy

**Keywords:** single-cell sequencing, myelodysplastic syndromes, acute myeloid leukemia, clonal hematopoiesis, immunotherapies, immune microenvironment

In the published article, there was an error in affiliation Number 1. Instead of “Department of Experimental Oncology, Istituto Europeo di Oncologia, Milan, Italy”, it should be “Department of Experimental Oncology, IRCCS Istituto Europeo di Oncologia, Milan, Italy”.

The authors apologize for this error and state that this does not change the scientific conclusions of the article in any way. The original article has been updated.

## Publisher’s note

All claims expressed in this article are solely those of the authors and do not necessarily represent those of their affiliated organizations, or those of the publisher, the editors and the reviewers. Any product that may be evaluated in this article, or claim that may be made by its manufacturer, is not guaranteed or endorsed by the publisher.

